# The Protective Role of Folic Acid in Biochemical and Histopathological Changes Induced by Azithromycin in the Livers of Pregnant Albino Rats

**DOI:** 10.3390/medicina61030415

**Published:** 2025-02-27

**Authors:** Safaa M. Hanafy, Soha S. Zakaria

**Affiliations:** 1 Department of Anatomy and Physiology, College of Medicine, Imam Mohammad Ibn Saud Islamic University (IMSIU), Riyadh 13317, Saudi Arabia; 2 Department of Biochemistry, College of Medicine, Imam Mohammad Ibn Saud Islamic University (IMSIU), Riyadh 13317, Saudi Arabia; sshehata@imamu.edu.sa

**Keywords:** folic acid, liver, pregnancy, azithromycin, albino rats

## Abstract

*Background and Objectives*: We evaluated the impact of the second-generation macrolide azithromycin on pregnant albino rats’ livers and assessed the protective role of folic acid. *Materials and Methods*: For this study, forty pregnant rats were treated and then examined. Forty pregnant albino rats were divided into four equal groups (I–IV). In Group I, each pregnant rat was gavaged with 1 mL distilled water on day 16 of pregnancy, then 0.5 mL from day 16 of pregnancy to day 20. In Group II, each pregnant rat was gavaged with 0.45 mL distilled water containing 9 mcg folic acid from day 17 of pregnancy to day 20. In Group III, each pregnant rat was gavaged with 1 mL distilled water containing 9 mg azithromycin on the 16th day, then 0.5 mL distilled water containing 4.5 mg azithromycin from day 17 of pregnancy to day 20. In Group IV, each pregnant rat was gavaged with 1 mL of distilled water containing 9 mg azithromycin and 0.45 mL distilled water containing 9 mcg folic acid on the 16th day, then 0.5 mL of distilled water containing 4.5 mg azithromycin and 0.45 mL distilled water containing 9 mcg folic acid from day 17 of pregnancy to day 20. At the end of the experiments, blood samples and the livers of the studied groups were subjected to biochemical, histological, and morphometric analysis. *Results*: Azithromycin induced pathological changes in the livers, as evidenced by disturbed lobular architecture, cytoplasmic vacuoles, deeply stained hepatic nuclei, and elevated liver enzymes. The co-administration of folic acid ameliorated most pathological changes. *Conclusions:* Azithromycin caused pathological alterations in the livers of pregnant rats, and the co-supplementation of folic acid with azithromycin is encouraged, to minimize these changes.

## 1. Introduction

The liver, the body’s primary metabolic detoxification organ, can be harmed by drugs, metabolites, and/or environmental toxins that enter the body [1]. Macrolides are among the most often prescribed antibiotics during pregnancy [2,3]. Azithromycin (AZM) is a member of the second generation of macrolides [4] that is prescribed for the treatment of infections during pregnancy because it has a good safety profile and strong antibacterial properties [5]. Azithromycin is useful in treating respiratory tract infections, tonsillitis, sinusitis, otitis media, skin and soft tissue infections, and other illnesses brought on by sensitive bacterial infections. [6,7]. In recent years, AZM has become more well known due to its potential as a treatment for COVID-19 infection rather than for its main antimicrobial effect [3,8]. As AZM is taken orally, it is primarily eliminated through biliary excretion. The half-life of AZM in humans is approximately 35–40 h following a 500 mg dose [9]. According to Ismael and Elsamman [10], the liver has the highest tissue concentration of AZM, followed by the kidneys, spleen, lungs, and heart. According to Salimi et al. [11], AZM has frequently caused stomach distress, diarrhea, nausea, vomiting, and abdominal pain as side effects. Additionally, recent studies have shown that AZM induces hepatotoxicity and increases the reactive oxygen species (ROS) level [12,13,14]. The oxidizing effect of ROS is actively counteracted by reduced glutathione (GSH). It is a tripeptide present in the cells of all tissues and is essential for both antioxidant defense and the control of processes that maintain cellular homeostasis [15]. MDA (Malondialdehyde) is a well-established biomarker of lipid peroxidation and oxidative stress. It is formed as a byproduct when ROS attack polyunsaturated fatty acids in cell membranes, leading to cellular damage and dysfunction. MDA levels provide insights into the extent of lipid peroxidation and oxidative damage [16].

Folic acid, sometimes known as vitamin B9, is a water-soluble vitamin. It is a micronutrient and an essential nutrient required for healthy human, growth, and development [17]. In both the liver and intestinal epithelial cells, dihydrofolate reductase sequentially reduces folic acid to produce dihydro- and tetrahydrofolate, which in turn yields 5-methyltetrahydrofolate, the most prevalent and physiologically active form of folic acid [18,19]. Numerous physiological processes depend on folic acid, particularly those involving methionine regeneration, nucleic acid synthesis, and single-carbon transfer reactions [20,21]. Folic acid has been shown to scavenge ROS, inhibit the activity of enzymes that produce ROS, restore the activity of antioxidant enzymes, and regulate lipid metabolism and oxidative stress. It effectively scavenges free radicals [22]. The current study aimed to evaluate how azithromycin adversely affects pregnant albino rats’ livers and assess the possible protective role of folic acid.

## 2. Materials and Methods

### 2.1. Drugs

Azithromycin was obtained from Pfizer, Giza, Egypt. It was accessible in the form of tablets of 250 mg each. The standard doses of azithromycin for the treatment of infection comprise 500 mg on day 1 followed by 250 mg daily from day 2 to day 5 [23]. The dose was adjusted according to the weight of the rat. According to Paget and Barnes’ formula [24], the calculated dose for an adult rat weighting approximately 200 g is 9 mg on the first day and then 4.5 mg/day from day 2 to day 5. Each tablet was dissolved in 28 mL distilled water and each rat was given 1 mL distilled water containing 9 mg Azithromycin on the first day and then 0.5 mL distilled water containing 4.5 mg Azithromycin from day 2 to day 5.

Folic acid was obtained from Mepaco, Cairo, Egypt. It was available in the form of tablets, each containing 500 micrograms. The dose for pregnant women is 500–600 micrograms/day, taken orally [23]. According to Paget and Barnes [24], the dose of folic acid per adult rat (weighing 200 g) was 9 micrograms/rat/day. Each tablet was dissolved in 25 mL of distilled water, and each rat was given 0.45 mL distilled water containing 9 micrograms folic acid.

### 2.2. Animals

#### 2.2.1. Animals Used in the Experiment

For the study, twenty male and forty female albino rats of the local strain, weighing between 190 and 200 g, were obtained. The animal care and experiments were conducted in accordance with the protocols approved by the Ethics Research Committee, Faculty of medicine, Tanta University (Approval code 2809/6/23). For twenty-four hours, each pair of virgin female rats was kept in a cage with a male rat. The vaginal plug’s appearance within the morning determined the first day of pregnancy [25]. Every pregnant rat was put in a separate cage until the end of the experiment.

#### 2.2.2. Experimental Design

Pregnant albino rats (*n* = 40) were divided equally into 4 groups (I, II, III, and IV). Each pregnant albino rat was gavaged for 5 days during the third trimester of pregnancy (the 16th, 17th, 18th, 19th, and 20th day). In Group I (the control), each pregnant albino rat was gavaged with 1 mL distilled water on day 16 and then 0.5 mL from day 17 through day 20. In Group II (the folic acid group), each pregnant albino rat was gavaged with 0.45 mL distilled water containing 9 mcg folic acid from day 16 to day 20. In Group III (azithromycin-treated), each pregnant albino rat was gavaged with 1 mL distilled water containing 9 mg azithromycin on day 16 and then 0.5 mL distilled water containing 4.5 mg azithromycin from day 17 through day 20. In Group IV (azithromycin-and-folic-acid- treated), each pregnant albino rat was gavaged with 1 mL of distilled water containing 9 mg azithromycin and 0.45 mL distilled water containing 9 mcg folic acid on day 16 and then 0.5 mL of distilled water containing 4.5 mg azithromycin and 0.45 mL distilled water containing 9 mcg folic acid from day 17 through day 20.

### 2.3. Blood Sample Analysis

Liver enzyme levels (AST and ALT) were measured in serum using a commercial assay kit, following the manufacturer’s instructions. The results were reported in IU/L, as per the kit specifications. The reference method cited for these measurements was Thomas’s method [26].

### 2.4. Histological Analysis

Large liver portions from every pregnant rat were gathered for each group. On the day following birth, rats were euthanized, a midline cut was made to reveal the abdominal cavity, and the livers were removed. All groups’ liver sections were used for light and electron microscopy analysis.

#### 2.4.1. Light Microscopy Analysis

Liver samples were immersed in 10% formal saline solution for three days before being dried in increasing ethanol concentrations of 70%, 90%, and 100% and cleaned with benzene. Paraffin wax was used to impregnate the specimens. A rotatory microtome was used to cut the paraffin squares into serial transverse segments with a thickness of 4 μm. An albumenized glass slide was used to mount each of the five progressive transverse paraffin sections. Hematoxylin and eosin were used to stain the subsequent slides obtained from each liver sample in order to illustrate the common hepatic architecture [27]. To illustrate the collagen fibers, Masson’s trichrome stain was used [28].

#### 2.4.2. Electron Microscopy Analysis

Very tiny liver fragments were preserved by immersing them in 5% glutaraldehyde in 0.1 M sodium cacodylate buffer at pH 7.3. After that, the samples were post-fixed in the same sodium cacodylate buffer using 1% osmium tetroxide. After that, the samples were cleaned, dehydrated, and embedded in epoxy resin [29]. The LKB ultratome was used to cut the semi-thin (1 μm thick) sections, which were then stained with toluidine blue and viewed under a light microscope. Uranyl acetate and lead citrate were used to stain the ultrathin (60 nm thick) sections after they were cut and placed on copper grids. A transmission electron microscope was used to examine the ultrathin sections (JEOL1010 EX II, Japan) at Mycology and Biotechnology Center, Cairo, Egypt.

### 2.5. Liver Homogenate Preparation and Biochemical Assessment of Oxidative Stress Markers

On the first day following delivery, liver samples were obtained from all groups of pregnant rats. To remove any red blood cells and clots, the samples were first perfused with a phosphate-buffered saline solution (PH7.4) that contained 0.16 mg/mL heparin. The tissues were centrifuged for 15 min at 4000 rpm after being homogenized in 5–10 milliliters of cold buffer per gram of tissue. MDA and GSH were measured in the supernatant. GSH was measured in accordance with Beutler et al. [30], and MDA (lipid peroxidation active product) was determined in accordance with Okhawa et al. [31]. The unit of measurement for reduced glutathione is mg/g tissue, while mmol/g tissue is the unit of measurement for malondialdehyde concentration. After being measured, the MDA and GSH means were tabulated and subjected to statistical analysis.

### 2.6. Morphometric Analysis

The surface area of collagen fibers was measured using a Leica Qwin 500 (England) image analysis computer system. From equally spaced locations along each serial section, ten Masson’s trichrome-stained slides were extracted from ten pregnant rats in each group. The collagen fiber surface area was then measured at 100× magnification in 10 fields on each slide. The collagen fibers’ cumulative average surface area in each subgroup was determined and statistically examined.

### 2.7. Statistical Analysis

The levels of ALT, AST, and collagen fibers in the adult groups were statistically analyzed using SPSS, version 26.0 statistical software. Tukey post hoc multiple-comparisons tests and analysis of variance (ANOVA) were used to analyze the data and determine statistical significance [32].

## 3. Results

### 3.1. Clinical Observations

- No maternal mortality was observed when comparing the mothers who received azithromycin to the other groups.
- No miscarriage was observed in the azithromycin-treated group compared to other groups.

### 3.2. Histopathological Analysis

#### 3.2.1. Light Microscopy

**Figure 1:**
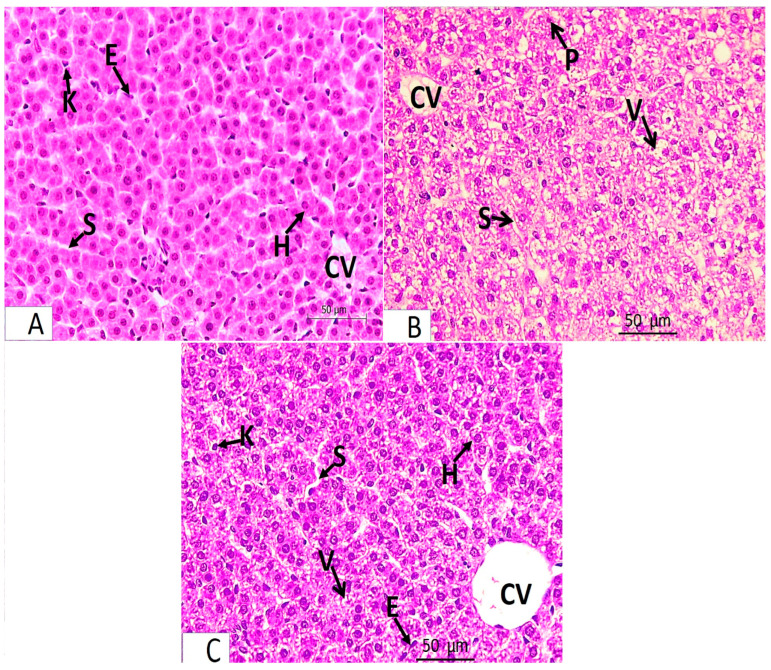
Light microscopy of H-E-stained liver sections from pregnant rats from Groups I–IV at ×400 magnification. Panel (**A**) represents Groups I and II (control and folic acid groups). Panel (**B**) represents Group III (the azithromycin-treated group). Panel (**C**) represents Group IV (folic-acid-and-azithromycin-treated group). (**A**) Hepatocytes (H) are arranged in cords radiating from the central vein (CV) and are separated by the blood sinusoids (S), which are lined by flat endothelial cells (E) and von Kupffer cells (K). (**B**) The hepatic cords are not radially arranged around the central vein (CV). They are separated by blood sinusoids (S). Most hepatocytes have a vacuolated cytoplasm with some nuclei displaced to the side of cells (V). Some of the hepatocyte nuclei are small and deeply stained (P). (**C**) Anastomosing hepatic cords radiating from the central vein (CV). The blood sinusoids (S) are lined with flat endothelial cells (E) and von Kupffer cells (K). Most hepatocytes appear normal (H). Few focal areas of vacuolated hepatocytes (V).

**Figure 2:**
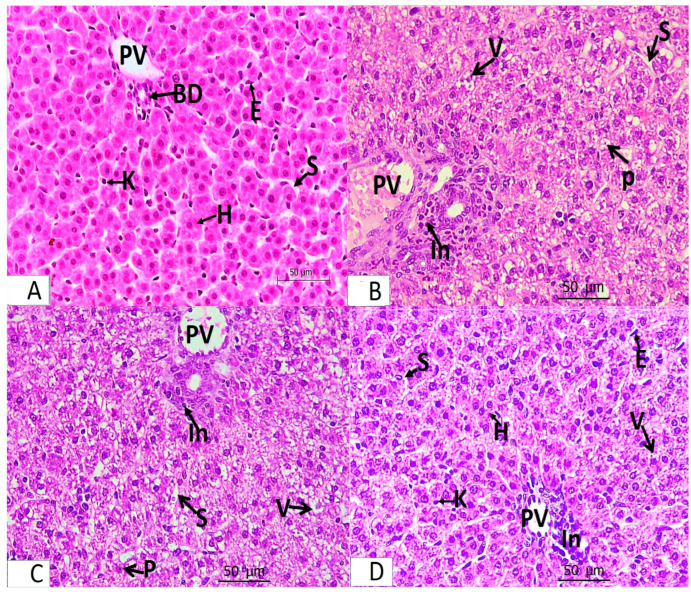
Light microscopy of H-E-stained liver sections from pregnant rats from Groups I–IV at ×400 magnification. Panel (**A**) represents Groups I and II (control and folic acid groups). Panel (**B**,**C**) represent Group III (azithromycin-treated group). Panel (**D**) represents Group IV (folic-acid-and-azithromycin-treated group). (**A**) Branches of the portal vein (PV) and bile duct (BD) are shown. The hepatocytes (H) are separated by blood sinusoids (S), which are lined with flat endothelial cells (E) and von Kupffer cells (K). (**B**,**C**) Most hepatocytes around the portal vein (PV) have a vacuolated cytoplasm (V). Some nuclei are small and deeply stained (P). The hepatocytes are separated by blood sinusoids (S); lymphocytic infiltration around the portal tract (In) is observed. (**D**) Portal vein (PV) and blood sinusoids (S) lined with flat endothelial cells (E) and von Kupffer cells (K). Most hepatocytes appear normal (H). Several focal areas of vacuolated hepatocytes and a slight infiltration of the portal tract (In) are observed.

**Figure 3:**
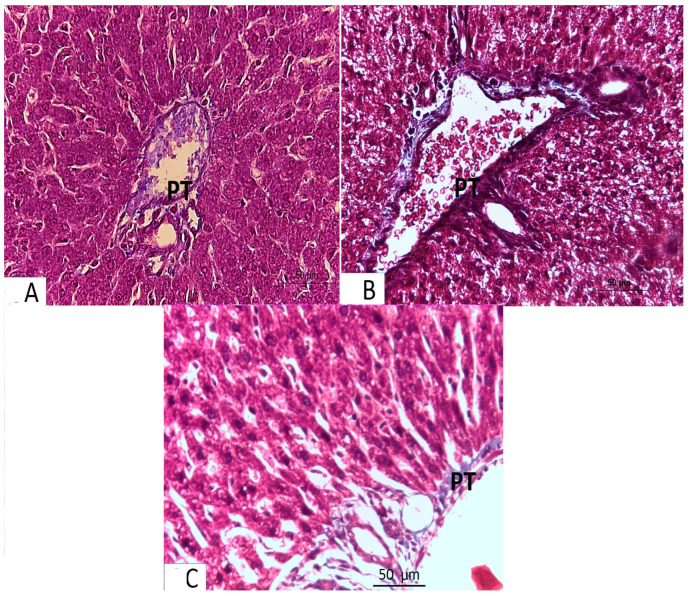
Light microscopy of Masson trichrome-stained liver sections from pregnant rats from Groups I–IV (×400 magnification). (**A**) Control and folic acid groups showing a normal distribution of collagen fibers around the portal tract (PT). (**B**) Azithromycin-treated group showing a marked increase in the level of collagen fiber distribution around the elements of the portal tract (PT). (**C**) Folic-acid-and-azithromycin-treated group showing a slight increase in collagen fiber distribution around the elements of the portal tract (PT).

1. The light microscopic analysis of stained liver sections (H–E) from the control group (Group I) and the folic acid group (Group II) showed no discernible differences between the two groups, so they were pooled together. It demonstrated that the liver was composed of hepatocyte cords that regularly radiated from the central vein and were divided by slit-shaped blood sinusoids lined with von Kupffer cells and flat endothelial cells (Figure 1A). The polyhedral hepatocytes had large vesicular basophilic nuclei and granular eosinophilic cytoplasm. One branch of the bile duct and one branch of the portal vein made up the portal tract (Figure 2A). Collagen fibers were normally distributed throughout the portal tract’s components and in the blood sinusoids’ wall, according to Masson’s trichrome staining (Figure 3A).
2. An analysis of the stained liver sections of the treated pregnant rats (the azithromycin-treated group) (H–E) revealed that the hepatic cords lacked the typical radial arrangement. Some of the blood sinusoids seemed clogged. The majority of hepatocytes contained cytoplasmic vacuoles with the nucleus displaced to the periphery; some of the nuclei were tiny and stained deeply (Figure 1B and Figure 2B,C). The portal tract showed lymphocytic infiltration (Figure 2B,C). Collagen fibers were more widely distributed around the portal tract’s components and in the blood sinusoids’ walls, according to Masson’s trichrome staining (Figure 3B).
3. Analysis of the H-E-stained liver sections of treated rats (folic-acid-and-azithromycin-treated) revealed that the majority of the hepatic cords had a radial arrangement around the central vein. Round vesicular nuclei and a granular eosinophilic cytoplasm characterized the majority of polyhedral hepatocytes. Cytoplasmic vacuoles were relatively few in hepatocytes (Figure 1C and Figure 2D). A slight increase in collagen fibers was observed around the portal tract’s components, as demonstrated by Masson’s trichrome staining (Figure 3C).

#### 3.2.2. Electron Microscopy

**Figure 4:**
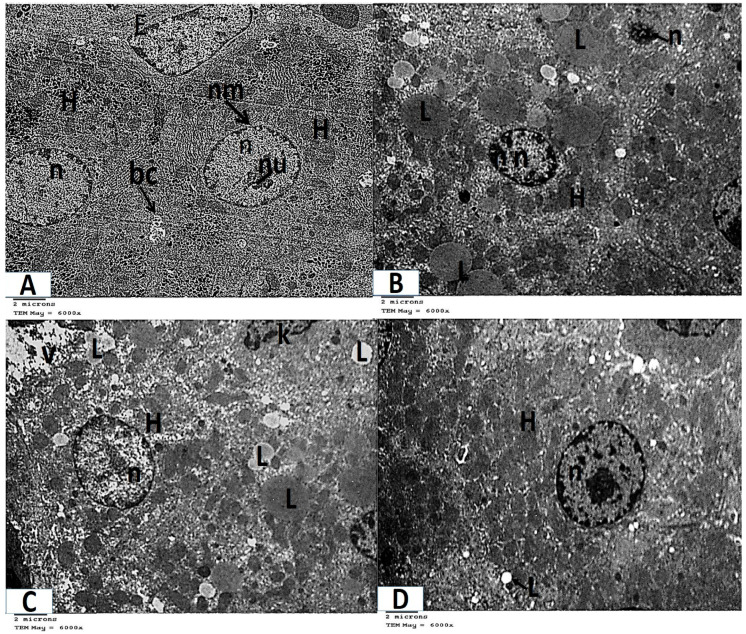
Photomicrographs of transmission electron microscopy of liver sections (**A**–**D**, TEM ×6000 magnification). (**A**) Control and folic acid groups (Group I and II) showing hepatocytes (H) with spherical regular nuclei (n) that have a nuclear envelope (nm) and nucleolus (nu). Notice the presence of flat endothelial cells (E) that line the blood sinusoid and bile canaliculus (bc). (**B**) Group III (azithromycin-treated), showing numerous lipid droplets (L) throughout the cytoplasm of the hepatocytes (H). Hepatocyte nuclei (n) appear shrunken with increased heterochromatin contents. (**C**) Group III (azithromycin-treated) showing lipid droplets (L) and vacuolated areas (V) throughout the cytoplasm of the hepatocytes (H). Hepatocyte nuclei (n) have a thick nuclear envelope. Part of a Kupffer (K) cell is noticed. (**D**) Group IV (folic-acid-and-azithromycin-treated) showing several lipid droplets (L) throughout the hepatocytes (H). The hepatocytes have an euchromatic nucleus (n).

**Figure 5:**
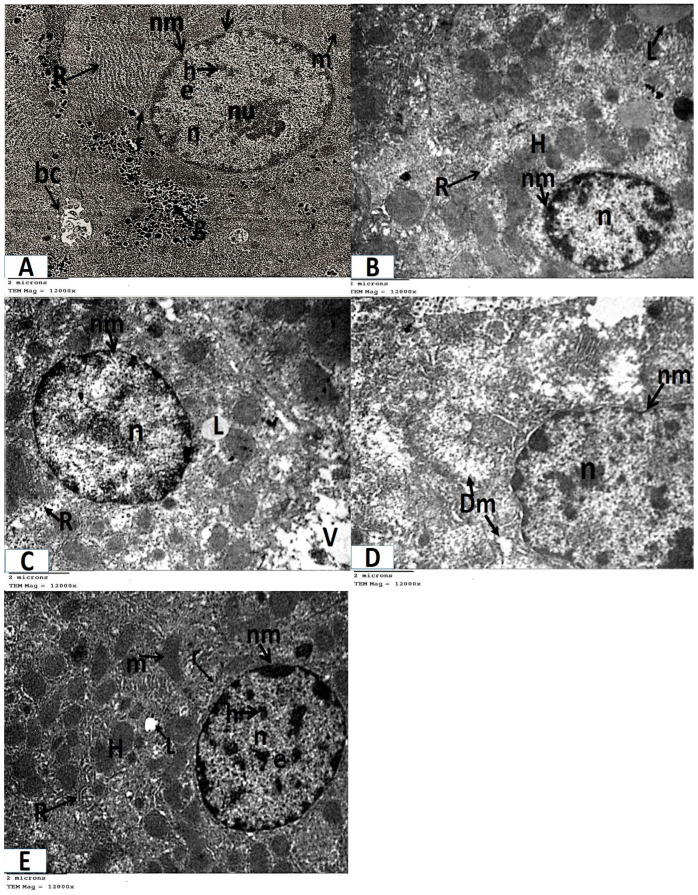
Photomicrographs of transmission electron microscopy of liver sections (A–E, TEM × 12,000 magnification). (**A**) Control and folic acid groups (Group I and II) showing that the hepatocyte has a nucleus (n), which is nearly rounded and has a distinct nuclear envelope (nm) with many nuclear pores (arrow). The nucleus contains heterochromatin (h), euchromatin (e), and a prominent nucleolus (nu). The cytoplasm contains mitochondria (m), ribosomes (r), a rough endoplasmic reticulum (R), and glycogen granules (g). Notice the presence of bile canaliculus (bc). (**B**) Group III (azithromycin-treated) showing lipid droplets (L) in the cytoplasm of the hepatocyte (H). The nucleus (n) appears shrunken with increased heterochromatin content and the nuclear envelope (nm) appears ill defined in several regions. Notice the fragmented rough endoplasmic reticulum (R). (**C**) Group III (azithromycin-treated) showing lipid droplets (L) in the cytoplasm of the hepatocyte. The nucleus (n) of the hepatocyte has a thick nuclear envelope (nm) with ill-defined nuclear pores in some regions. The cytoplasm contains vacuolated areas (V) and a fragmented rough endoplasmic reticulum (R). (**D**) Group III (azithromycin-treated) showing a nearly normal-sized nucleus (n) with an irregular nuclear envelope (nm). The cytoplasm contains mitochondria (Dm) with destroyed or lost cristae. (**E**) Group IV (folic-acid-and-azithromycin-treated), showing hepatocytes (H) with a nucleus (n) that is nearly rounded. The nucleus has a distinct nuclear envelope (nm) with many nuclear pores. The nucleus contains heterochromatin (h) and euchromatin (e). The cytoplasm contains mitochondria (m), ribosomes (r), a few lipid droplets (L), and a rough endoplasmic reticulum (R).

1. Analysis of ultrathin sections of the control and folic acid groups (Groups I and II, respectively) revealed negligible histological differences, so they were combined. The hepatocytes were found to have rounded nuclei with distinct nuclear envelopes that contained numerous nuclear pores. Both euchromatin and heterochromatin were present in the nuclei, along with noticeable nucleoli. Numerous cellular organelles, primarily mitochondria, which had an oval or rounded appearance, free ribosomes, a rough endoplasmic reticulum, which had parallel cisternae studded with ribosomes, and glycogen granules, which were dispersed or clustered, were all found in the cytoplasm (Figure 4A and Figure 5A).
2. Ultrathin sections of Group III (the azithromycin-treated group) revealed histopathological alterations in the hepatocytes. The most obvious result was the accumulation of many different-sized lipid droplets that were both electron-dense and electron-lucent throughout the hepatocytes’ cytoplasm. Some nuclei were small nuclei with thick nuclear envelopes and poorly defined nuclear pores. Other nuclei had high concentrations of electron-dense chromatin and were shrunken. The cytoplasm had a fragmented and dispersed rough endoplasmic reticulum, degenerated mitochondria with lost cristae, and vacuolated areas (Figure 4B,C and Figure 5B–D).
3. Analysis of extremely thin liver sections from Group IV (those treated with folic acid and azithromycin) revealed that the hepatocytes had rounded nuclei with a distinct nuclear envelope and nuclear pores. Both chromatin and heterochromatin were present in the nuclei, along with noticeable nucleoli. Ribosomes, mitochondria, glycogen granules, and parallel cisternae of the rough endoplasmic reticulum were all found in the cytoplasm. Lipid droplets were few (Figure 4D and Figure 5E).

### 3.3. Liver Indices

- A statistical analysis of the mean ALT and AST levels in Groups I, II, III, and IV revealed that Groups III and IV had significantly higher levels (*p* < 0.05) than Groups I and II. Additionally, it showed a significant (*p* < 0.05) decline in Group IV relative to Group III (Table 1).

### 3.4. Assessment of Oxidative Stress Markers

A statistical analysis of the mean GSH levels in the livers of the studied groups showed that Group III (azithromycin-treated) had a significant (*p* < 0.05) decrease in GSH levels compared to Groups I and II. Additionally, it showed that Group IV (azithromycin-and-folic-acid-treated) had a significant (*p* < 0.05) increase in comparison to Group III (Table 1). The mean MDA levels in the livers of Groups I–IV were statistically analyzed, and Group III showed a significant (*p* < 0.05) increase in MDA levels compared to Groups I and II. Additionally, it was shown that Group IV had a significant (*p* < 0.05) decline in comparison to Groups I and III (Table 1).

### 3.5. Morphometric Analysis

A statistical analysis of the mean collagen fiber surface area levels in Groups I, II, III, and IV revealed that Groups III and IV had significantly higher levels (*p* < 0.05) than Groups I and II. Additionally, a significant (*p* < 0.05) decline in Group IV relative to Group III was found (Table 1).

## 4. Discussion

Azithromycin is a common treatment for infections during pregnancy because it has a good safety profile and strong antibacterial properties [5]. However, recent studies have shown that it may be hepatotoxic [14]. In this study, we investigated the effects of azithromycin on the liver of pregnant rats and evaluated the protective potential of folic acid.

In the current study, the examination of pregnant rats’ livers in the azithromycin-treated group revealed that the majority of hepatocytes contained cytoplasmic vacuoles with the nucleus displaced to the periphery; some of the nuclei were tiny and stained deeply. Additionally, the accumulation of many different-sized lipid droplets throughout the hepatocytes was observed by electron microscopy. These findings were consistent with those of Paulose et al. [33] and Usadadia et al. [34], who used biochemical and histopathological methods to examine the toxicity induced by azithromycin in experimental rats. They found that administering azithromycin (30 mg/kg b.wt.) caused a significant amount of liver damage, as evidenced by severe sinusoidal hemorrhages, congestion, vacuolar degeneration, fatty changes, and hepatic cord disruption in the liver. Also, Xu et al. [14] incubated mouse primary hepatocytes with azithromycin (0, 1, 5, 10, 20, 50, 100, 200, 500, and 1000 μmol·L^−1^) for 6, 12, 24, and 36 h. They discovered that, in a dose-and time-dependent manner, azithromycin dramatically reduced cell activity. Under a microscope, many floating dead cells were visible.

Our study exhibited that the portal tract of the liver-stained sections of the azithromycin-treated group showed lymphocytic infiltration. These data were consistent with Abdelaziz et al. [35], who found that the examination of serial liver sections from rats treated with azithromycin (6 mg/kg b.wt. for 10 days) showed a distinctive hepatotoxic histopathological alteration, as evidenced by significant portal, perivascular, and interstitial lymphocyte infiltration.

Our data exhibited that the collagen fibers of stained liver sections from the azithromycin-treated group were more widely distributed around the portal tract’s components and in the blood sinusoids’ walls compared to the control. These results were consistent with those of Abd El-Naeem et al. [36] and Abd El-Kader [37], who used Masson’s trichrome staining to investigate the cardiotoxic effect of azithromycin (30 mg/kg/day) on adult male rats. They showed that the azithromycin-treated group had more collagen fibers than the control group.

In the current study, ultrathin sections from the group treated with azithromycin revealed that some nuclei were shrunken and had high concentrations of electron-dense chromatin. The cytoplasm had a fragmented rough endoplasmic reticulum, enlarged or degenerated mitochondria with lost cristae, and vacuolated areas. Our findings were consistent with those of Jiang et al. [38], who showed that azithromycin caused DNA damage, mitochondrial toxicity, and reactive oxygen species (ROS) in human mammary epithelial cells and primary fibroblasts. Also, Jassab et al. [39] found significant DNA degradation in their group that received azithromycin at a concentration of 30 mg/kg.

We found that the mean ALT and AST levels in the azithromycin-treated group showed significantly higher levels (*p* < 0.05) than the control group. These results were consistent with those of Usadadia et al. [34] and Abdelaziz et al. [35], who observed that oral azithromycin administration markedly raised ALT and AST activities.

Additionally, our data provided evidence for an altered redox status as the azithromycin treated group had a significant decrease in GSH levels compared to Groups I and II. Also, the mean MDA levels showed a significant increase in the azithromycin-treated group compared to Groups I and II. These results might explain the histopathological changes observed on the basis of the disturbance of the oxidant–antioxidant ratio. Xu et al. [14] explained the degenerative changes induced by azithromycin on the basis of ROS elevation. Also, Abdelaziz et al. [35] observed that oral azithromycin administration for 10 consecutive days in male rats resulted in a significantly higher level of MDA and a significantly lower level of GSH when compared to the control. An elevated MDA level is a sign of oxidative stress and a good indicator of lipid peroxidation [40]. GSH is a soluble antioxidant that lowers ROS levels, and it is found in the mitochondria, nucleus, and cytoplasm. The drop in the level of GSH is a sign of oxidative stress [41]. This oxidative stress interfered with the liver’s normal cellular functions [42].

Notably, our data showed that the livers of rats from the azithromycin-and-folic-acid-treated group restored their architecture. The majority of the hepatic cords had a radial arrangement around the central vein. Round vesicular nuclei and granular eosinophilic cytoplasm characterized the majority of polyhedral hepatocytes. Cytoplasmic vacuoles were relatively few in hepatocytes. These findings were confirmed by a significant reduction in the level of liver enzymes as compared to the azithromycin-treated group. Additionally, Group IV showed significant elevation in mean GSH levels and a reduction in mean MDA levels compared to those of Group III. These findings were consistent with a study [43] in male Balb/c mice. In this study, the impact of folic acid (0.01 g FA/kg) on liver damage caused by isoniazid (0.66 g INH/kg) was examined. The authors noticed that folic acid treatment lessened liver necrosis and dramatically lowered the alanine aminotransferase level. They also mentioned that folic acid supplementation decreased CYP2E1’s mRNA and protein expression, which in turn decreased the expression of F4/80 mRNA and liver MDA [41]. Also, Zhang et al. [44] investigated the possible protective role of folic acid in alcohol-induced hepatic injury in male C57BL/6J mice. They observed that folic acid (5.0 mg/kg/day/10 weeks) improved alcohol-induced hepatic lipid deposition and inflammation and reduced serum levels of ALT and AST in male C57BL/6J mice. In addition, Khowaja et al. [45] reported that folic acid treatment (2.5 mg/kg-bw for 4 weeks) is very effective in preventing and reversing the hepatic serum marker levels and hepatic histological alterations brought on by fluoride administration in Wistar albino rats. Folic acid supplementation has been demonstrated to scavenge oxygen free radicals, regulate the activity of enzymes that produce oxygen free radicals, and replenish the activity of antioxidant enzymes [46]. Asbaghi et al. [47] stated that folic acid may greatly enhance the markers of antioxidative defense system by raising serum levels of GSH and the total antioxidant capacity while lowering MDA levels.

In the present work, the examination of pregnant rats’ livers in the azithromycin-and-folic-acid-treated group revealed that fine collagen fibers were distributed around the portal tract’s components, as demonstrated by Masson’s trichrome staining. Similar results were obtained by Xin et al. [48], who stated that folic acid treatment (15 mg/kg/d) for 8 weeks in rats reduced the severity of fibrosis caused by a high-fat diet (16 weeks).

### Study Limitations

1. The parameters for Gamma-glutamyl transferase (γ-GT) and alkaline phosphatase were not measured
2. The exact number of births recorded on each specific day of pregnancy (21, 22, and 23) was not documented during the experiment.

## 5. Conclusions

Azithromycin caused pathological alterations in the livers of pregnant rats, as shown by the accumulation of lipid droplets, deeply stained hepatic nuclei, cytoplasmic vacuoles, disrupted lobular architecture, elevated enzymes, and a disturbed oxidant–antioxidant ratio. Folic acid co-administration reduced the majority of pathological changes. Thus, if azithromycin is prescribed to pregnant women, it is advised that they take folic acid in supplements along with it. Further research is required to study other possible mechanisms through which folic acid elicits its protective role. Additionally, further research is required to study the effect of administrating pregnant rats with folic acid and azithromycin on their offspring.

## Figures and Tables

**Table 1:** Means and standard deviations for ALT, AST, collagen surface area, and GSH and MDA in the livers of Groups I–IV.

Group	Group I	Group II	Group III	Group IV	ANOVA	
Variable					F-Value	*p*-Value
ALT (IU/L)	16.6 ± 1.64	17.5 ± 1.9	42.2 ± 3.7 ^a b^	25.2 ± 2.2 ^a b c^	212.8	<0.0001 *
AST (IU/L)	12 ± 1.15	12.3 ± 1.4	31.3 ± 2.79 ^a b^	19.8 ± 1.0328 ^a b c^	268.5	<0.0001 *
Collagen surface area	8.826 ± 1.62	9.53 ± 1.35	19.89 ± 1.81 ^a b^	13.7 ± 1.41 ^a b c^	105.4	<0.0001 *
GSH (mg/g)	1.37 ± 0.156	1.192 ± 0.06	0.73 ± 0.12 ^a b^	1.156 ± 0.345 ^c^	17.73	<0.0001 *
MDA (mmol/g)	21.92 + 0.81	22.45 + 0.52	35.6 + 1.74 ^a b^	23.63 + 0.87 ^a c^	355.71	<0.0001 *

Tukey’s post hoc test was used after one-way ANOVA to examine variations in ALT, AST, collagen fiber surface area, GSH, and MDA in Groups I–IV. At *p* < 0.05, values are considered statistically significant. ^a^ statistically significant difference from I; ^b^ statistically significant difference from II; and ^c^ statistically significant difference from III. * means significant differences between the groups.

## Data Availability

All data supporting the findings of this study are available upon reasonable request.
